# 711 Neuropathic Pain in Burn Patients - A Common Problem with Little Literature: A Systematic Review

**DOI:** 10.1093/jbcr/irae036.255

**Published:** 2024-04-17

**Authors:** Eloise Stanton, Paul Won, Artur Manasyan, Sandeep Gurram, Anait Shirinian, Justin Gillenwater, Haig A Yenikomshian

**Affiliations:** Keck School of Medicine of USC, Los Angeles, CA; Keck School of Medicine, Los Angeles, CA; Keck School of Medicine, University of Southern California, Los Angeles, CA; Los Angeles General, Van Nuys, CA; Keck Medicine of USC, Los Angeles, CA; University of Southern California, Los Angeles, CA; Keck School of Medicine of USC, Los Angeles, CA; Keck School of Medicine, Los Angeles, CA; Keck School of Medicine, University of Southern California, Los Angeles, CA; Los Angeles General, Van Nuys, CA; Keck Medicine of USC, Los Angeles, CA; University of Southern California, Los Angeles, CA; Keck School of Medicine of USC, Los Angeles, CA; Keck School of Medicine, Los Angeles, CA; Keck School of Medicine, University of Southern California, Los Angeles, CA; Los Angeles General, Van Nuys, CA; Keck Medicine of USC, Los Angeles, CA; University of Southern California, Los Angeles, CA; Keck School of Medicine of USC, Los Angeles, CA; Keck School of Medicine, Los Angeles, CA; Keck School of Medicine, University of Southern California, Los Angeles, CA; Los Angeles General, Van Nuys, CA; Keck Medicine of USC, Los Angeles, CA; University of Southern California, Los Angeles, CA; Keck School of Medicine of USC, Los Angeles, CA; Keck School of Medicine, Los Angeles, CA; Keck School of Medicine, University of Southern California, Los Angeles, CA; Los Angeles General, Van Nuys, CA; Keck Medicine of USC, Los Angeles, CA; University of Southern California, Los Angeles, CA; Keck School of Medicine of USC, Los Angeles, CA; Keck School of Medicine, Los Angeles, CA; Keck School of Medicine, University of Southern California, Los Angeles, CA; Los Angeles General, Van Nuys, CA; Keck Medicine of USC, Los Angeles, CA; University of Southern California, Los Angeles, CA; Keck School of Medicine of USC, Los Angeles, CA; Keck School of Medicine, Los Angeles, CA; Keck School of Medicine, University of Southern California, Los Angeles, CA; Los Angeles General, Van Nuys, CA; Keck Medicine of USC, Los Angeles, CA; University of Southern California, Los Angeles, CA

## Abstract

**Introduction:**

The prevalence of neuropathic pain (NP) in burn patients is reported in the literature to be as high as 80%1. Given the complexity of NP in burn patients and the wide range of treatments available, a systematic review of the literature is warranted to summarize our current understanding of management and treatment of NP in this population.

**Methods:**

This systematic review was conducted according to the Preferred Reporting Items for Systematic Reviews and Meta-Analysis (PRISMA) guidelines. The following databases were queried to identify relevant articles: PubMed, Cochrane, Embase, Scopus, Ovid, and Web of Science. The main outcome measures were incidence and management of NP. Secondary outcomes included risk factors for NP.

**Results:**

Included articles presented findings from 11 different countries, capturing outcomes for 4,366 patients. Risk factors for chronic pain in burn patients were identified, including older age, alcohol and substance abuse, current daily smoking, greater % total body surface area burns (TBSA), and longer hospitalizations. Pharmacologic treatments included gabapentin/pregabalin (n=7), ascorbic acid (n=1), and lidocaine (n=1). Overall, the studies showed varied results regarding the efficacy of pharmacological treatments. While certain studies demonstrated gabapentanoids to be effective in reducing neuropathic symptoms, others found conflicting results. With regards to non-pharmacologic treatments, electroconvulsive therapy (n=1), electropuncture (n=1), nerve release/reconstruction (n=2), and somatosensory feedback rehabilitation (n=1) were used and demonstrated promise in reducing pain intensity and improving functionality.

**Conclusions:**

Despite NP afflicting the majority of burn patients long after their injury, this systematic review demonstrates that there is insufficient evidence on the pathophysiology, outcomes, and risk factors in NP, as well as the efficacy of various therapies. Future prospective and randomized studies evaluating the etiology of these factors can substantially improve our treatment strategies. This can allow for the development of well-delineated and evidence-based protocols in NP management in hopes of improving quality of life and both psychological and physical function in burn patients.

**Applicability of Research to Practice:**

Neuropathic pain is a common cause of chronic morbidity in burn patients, often lasting for years and causing limitations in regard to quality of life and function, yet there is currently a lack of understanding in regard to optimal treatment regimens for this devastating condition. The current review summarizes our knowledge of existing treatment modalities and identifies gaps that can help guide future research.

